# Supplementary material for: Comparative multiomics analysis of cell physiological state after culture in a basket bioreactor
Source: Sci Rep. 2022 Nov 23;12:20161. doi: 10.1038/s41598-022-24687-4 (PMC9686226; doi:10.1038/s41598-022-24687-4)
Supplement: Supplementary file 1 — Supplementary Information 1. [file 41598_2022_24687_MOESM1_ESM.zip › raw data/Proteomic raw data/6.KEGGenrichment/EG.vs.CG/ALL/pathway/src/04110.html]

map04110


- K02209, K02210, K02212, K02540, K02541, K02542

- Up regulated proteins


A0A0D9RQN8(Description:DNA replication licensing factor MCM4;Gene:MCM4;log2FC:1.0247)


A0A0D9RKU0(Description:DNA replication licensing factor MCM3;Gene:MCM3;log2FC:1.0877)

- K02212

- Up regulated proteins

A0A0D9RQN8(Description:DNA replication licensing factor MCM4;Gene:MCM4;log2FC:1.0247)

- K02541

- Up regulated proteins

A0A0D9RKU0(Description:DNA replication licensing factor MCM3;Gene:MCM3;log2FC:1.0877)

- K04802

- Up regulated proteins

A0A0D9RLR8(Description:Proliferating cell nuclear antigen;Gene:PCNA;log2FC:1.0975)

- K06636

- Up regulated proteins

A0A0D9RJF7(Description:Structural maintenance of chromosomes protein 1A;Gene:SMC1A;log2FC:1.3605)

- K06618

- Up regulated proteins

A0A0D9RYS3(Description:Uncharacterized protein;Gene:RB1;log2FC:1.1115)

- K06618

- Up regulated proteins

A0A0D9RYS3(Description:Uncharacterized protein;Gene:RB1;log2FC:1.1115)

- K06618

- Up regulated proteins

A0A0D9RYS3(Description:Uncharacterized protein;Gene:RB1;log2FC:1.1115)

- K06670

- Up regulated proteins

A0A0D9REV4(Description:Uncharacterized protein;Gene:RAD21;log2FC:1.4233)

Close
